# Feeling Bad and Looking Worse: Negative Affect Is Associated with Reduced Perceptions of Face-Healthiness

**DOI:** 10.1371/journal.pone.0107912

**Published:** 2014-09-26

**Authors:** Laura Mirams, Ellen Poliakoff, Elizabeth H. Zandstra, Marco Hoeksma, Anna Thomas, Wael El-Deredy

**Affiliations:** 1 School of Psychological Sciences, University of Manchester, Manchester, United Kingdom; 2 Unilever R&D, Vlaardingen, The Netherlands; 3 Unilever R&D, Port Sunlight, United Kingdom; Brock University, Canada

## Abstract

Some people perceive themselves to look more, or less attractive than they are in reality. We investigated the role of emotions in enhancement and derogation effects; specifically, whether the propensity to experience positive and negative emotions affects how healthy we perceive our own face to look and how we judge ourselves against others. A psychophysical method was used to measure healthiness of self-image and social comparisons of healthiness. Participants who self-reported high positive (*N* = 20) or negative affectivity (*N* = 20) judged themselves against healthy (red-tinged) and unhealthy looking (green-tinged) versions of their own and stranger’s faces. An adaptive staircase procedure was used to measure perceptual thresholds. Participants high in positive affectivity were un-biased in their face health judgement. Participants high in negative affectivity on the other hand, judged themselves as equivalent to less healthy looking versions of their own face and a stranger’s face. Affective traits modulated self-image and social comparisons of healthiness. Face health judgement was also related to physical symptom perception and self-esteem; high physical symptom reports were associated a less healthy self-image and high self-reported (but not implicit) self-esteem was associated with more favourable social comparisons of healthiness. Subject to further validation, our novel face health judgement task could have utility as a perceptual measure of well-being. We are currently investigating whether face health judgement is sensitive to laboratory manipulations of mood.

## Introduction

Rather than being a direct reflection of reality, perception is based on an interpretation of incoming sensory information [1]. Emotions can drive attentional and interpretational biases, leading to distortions in perception. Someone who is anxious about their health, for example, might pay attention to ambiguous bodily sensations, be biased to interpret these sensations as symptoms of illness and as a result, perceive themselves to be less healthy than they are in reality [2]. The purpose of the current study was to investigate whether differences in the propensity to experience positive and negative emotions affects not only how healthy we feel, but also how healthy we perceive our own face to look and how we judge ourselves against others.

Positive and negative affectivity are dispositional traits defined by tendencies to experience a range of pleasant or unpleasant emotions [3]. A state of high positive affect is characterised by pleasant feelings such as enthusiasm and alertness; with low positive affect associated with feelings of sadness and lethargy. A state of high negative affect is characterised by feelings of psychological distress, such as nervousness and irritability, with low negative affect associated with feelings of calmness and serenity. Low positive affect and high negative affect are considered to be independent dimensions and distinguishing features of depression and anxiety, respectively [4]. Individuals high in negative affectivity consistently report more physical symptoms and illnesses than individuals low in negative affectivity [5], [6] despite not differing in their objectively measured health [7].

Positive and negative affectivity are thought to contribute to subjective well-being, i.e., our sense, or perception of how we feel in general [8] and are also linked with more specific self-perceptions. For example, self-reported experience of positive affect in everyday life is associated with a positive self-concept (high self-esteem), whereas self-reported experience of negative affect is associated with a negative self-concept, or low self-esteem [9]. Most people in the general, adult population experience more positive affect than negative affect [10] and have a positive self-concept [11]. Generally, people are positively biased in their evaluations of self-related stimuli. They prefer the letters in their own name [12], for example, and are faster to categorise self and positive words using the same response key, than they are to categorise self and negative words using the same response key [13]. These positivity biases are taken to provide implicit (i.e., indirect, non-conscious) indicators of self-esteem, distinct from explicit (i.e., consciously reported) self-esteem [13]. Indeed, implicit and explicit self-esteem are often differentially related to other variables [9], [14].

Positive and negative affect may also affect how we perceive concrete, observable aspects of ourselves. Perhaps as a result of harbouring positive feelings towards the self, healthy females tend to underestimate their body size [15]. What is more, there is evidence that healthy people perceive their own faces to look more attractive and trustworthy than they do in reality. Epley and Whitchurch [16] morphed photographs of participants’ faces with images of attractive and unattractive composite faces resulting in a set of faces varying in attractiveness. Participants were more likely, and faster, to select an attractively enhanced face version as their own out of line-ups containing their original and morphed image. Individuals with higher scores on two implicit measures of self-esteem had more elevated perceptions of self-attractiveness. Explicit (self-reported) self-esteem, however, was not associated with enhancement in recognition; Epley and Whitchurch suggested that automatic positive associations to the self, rather than more deliberate and controlled assessments of the self, might have driven their enhancement effects. Verosky & Todorov [17] found a similar enhancement effect when participant’ faces were morphed with trustworthy and untrustworthy prototype faces; participants viewed themselves as more similar to trustworthy looking faces, and less similar to untrustworthy looking faces. Following this, Farmer, McKay and Tsakiris [18] found that participants view themselves as more similar to a person who has previously displayed trustworthy behaviour and less similar to a person who previously displayed untrustworthy behaviour.

Self-enhancement is thought to maintain a positive self-concept [19], but varies between individuals [20]. There is reason to think that individuals characterised by high negative affectivity might lack the self-image enhancement effects characteristic of happier people and even perceive themselves to look worse than they do in reality. Females with eating disorders, for example, who report high levels of negative affect [21], [22] tend to overestimate their body-size [15]. Individuals with symptoms of body dysmorphia, who feel negatively about their appearance, have also shown altered perceptions of their self-image compared to controls [23]. In one study [23] patients with body dysmorphia made more accurate judgements about their disliked body parts (the size of their nose) compared to patients without body dysmorphia. In addition, students who self-report symptoms of body dysmorphia have shown reduced perceptions of self-attractiveness [24]. Clerkin and Teachman [24] used a similar morphing procedure and self-recognition paradigm to Epley & Whitchurch [16]. They found that students with symptoms of body dysmorphia tended to rate unattractive versions of their face as more likely to be their own than attractive versions, whereas students without symptoms of body dysmorphia showed the opposite pattern (i.e., an enhancement, rather than derogation effect). There were no between-group differences in self-recognition in this study, however. The majority of participants were able to accurately identify their un-altered image from line-ups containing their original photograph and the morphed images.

The main aim of the current study was to further investigate individual differences in perceptions of self-image. Given the links between well-being, affectivity and subjective perceptions of health [5], [8] we investigated, first, whether the enhancement effects characteristic of happy people are also evident when skin tone, rather than facial symmetry is manipulated. Red skin colouration, associated with the presence of oxygenated blood, is linked with cardiovascular fitness and human sexuality [25]. Accordingly, increasing the amount of redness in the skin increases perceived healthiness and attractiveness of human faces [26]. Face health judgement has high ecological validity as an indicator of well-being; we often use our facial image as a cue to healthiness. If we can see some colour in our cheeks, we might feel healthier than if we look pale, for example. We investigated whether the reverse might also be true; when people feel good, do they perceive themselves to look healthier than they do in reality? Secondly, we investigated whether individuals characterised by generalised negative affectivity might lack this enhancement effect, or perceive themselves to look less healthy than they do in reality.

We manipulated healthiness of self-image by altering the skin tone in photographs of participants’ faces. We added or subtracted the amount of redness in photographs of participants’ faces, to produce pink and green-tinged versions that varied in how healthy they looked (see Figures for examples). Altering skin tone, rather than morphing participants’ photographs with attractive and unattractive composite faces, meant that faces differed only in how healthy they looked (which in turn affects attractiveness), but not in the degree to which they looked similar or dissimilar to the participant.

To measure perceptions of self-image we developed a novel face health judgement task using psychophysical methods. To estimate perceptual thresholds (i.e., estimate which face version roughly corresponded to participants internal representation of self-image), we used an adaptive staircase procedure [27] and compared perceptual thresholds between participants characterised by high positive versus high negative affectivity. The former participants were expected to perceive themselves as looking healthier than their original photograph. The latter participants were expected to lack this enhancement effect (perceive themselves as looking approximately as healthy as their original photograph), or show a derogation effect (perceive themselves as looking less healthy than their original photograph).

Our second aim was to investigate whether positive and negative emotionality affects how people judge self-healthiness against others. Evidence suggests that emotions affect how we judge ourselves against others [28], [29] and in turn, that such self-other comparisons shape our self-evaluations [30]–[32]. Whereas individuals with high self-esteem tend to evaluate themselves favourably against others, which increases positive affect, individuals with low self-esteem and depressed individuals, evaluate themselves unfavourably in relation to others, which increases negative affect [29]. Self-other comparisons also affect how individuals perceive their own faces. Using an adapted version of Epley & Whitchurch’s face recognition paradigm, Zell & Balcetis [33] found that after viewing same-gender attractive models, students rated themselves as less attractive and selected a less attractive version of their face as their own out of a line-up containing their original photograph among attractive and unattractive morphs. After viewing opposite-gender attractive models, unattractive same-gender peers or landscapes, participants rated themselves as more attractive and showed enhancement in self-recognition (i.e., selected a more attractive version of their face out of the line-up). We hypothesised that participants characterised by high positive affect would be likely to evaluate themselves favourably in comparison to others and as a result, judge themselves as equivalent to healthier looking versions of a stranger’s face. We expected participants characterised by high negative affect, on the other hand, to evaluate themselves unfavourably against others and as a result, judge themselves as equivalent to less healthy looking versions of a stranger’s face.

To address these aims, participants completed two different versions of the face health judgement task. In one version, participants judged how they felt compared to healthy and unhealthy looking versions of their own face (i.e., ‘how do I feel compared to this version of my own face?’). In the other version, participants judged how they felt compared to healthy and unhealthy looking versions of a stranger’s face (‘how do I feel compared to this version of a stranger’s face?’). Whereas the former version of the task was intended to measure self-image, the latter was intended to measure social comparisons; that is; whether people see themselves as equivalent to healthy or unhealthy looking versions of a stranger’s face (rather than measuring how healthy they perceived a stranger’s face to look).

We also investigated the relationship between face health judgement, subjective perceptions of health and self-esteem. We predicted subjective perceptions of health to be positively related to healthiness of self-image and stranger FHJ. We also expected that people with higher self-esteem would show more favourable face health judgement. Epley and Whitchurch found self perception to be related to implicit, but not explicit self-esteem. However, as face health judgment is a more deliberative and controlled process compared to simple recognition, we included an explicit, as well as implicit measure to determine whether face health judgement is related to conscious or non-conscious indicators of self-esteem.

In sum, we set out to investigate how positive and negative emotionality affect perceptions of self-image and how people judge self-healthiness against others, by developing a novel FHJ task. Using this paradigm, we demonstrated individual differences in perceptions of self-healthiness.

## Method

### Ethics statement

The experiment was approved by the University of Manchester Research Ethics Committee, Manchester, UK. Informed written consent was obtained prior to the study from all participants.

### Participants and recruitment

An advertisement for participants with normal or corrected to normal vision, without colour blindness, was placed on the University of Manchester research volunteering website. The advertisement included a link to an online pre-screen survey which included the Positive and Negative Affect Schedule (PANAS) [4]. The PANAS consists of a list of ten positive and ten negative feelings and emotions (e.g., active, determined, excited, afraid, distressed, and irritable) and respondents were instructed to rate the extent to which they had felt each feeling/emotion during the past few weeks on a scale from one (very slightly or not at all) to five (extremely). Scores on the positive and negative affect subscales range from ten to fifty, with high scores indicating high experience of positive/negative affect. The PANAS has good construct validity and test-retest reliability and this version provides an indication of dispositional affectivity [4], [10].

Out of 140 respondents, individuals who scored in the upper quartile of the positive affect scale (>39) and in the middle (17–26) or lower (<17) quartile on the negative affect scale, *N* = 34) and who obtained scores in the upper quartile of the negative affect scale (>26) and in the middle (30.75–39) or lower (<30.75) quartile on the positive affect scale, *N* = 32) were invited to take part in the main phase of the study. The final sample consisted of forty participants (aged 18–53); twenty in the high positive affect group (15 female, *M* age = 24.60, *SD* = 8.26, *M* positive affect score = 40.70, *M* negative affect score = 17.20) and twenty in the high negative affect group (17 female, *M* age = 23.85, *SD* = 6.05, *M* positive affect score = 30.10, *M* negative affect score = 34.55). In the positive affect group, 85% of participants were Caucasian, 5% were South Asian and 10% were Black. In the negative affect group, 75% of participants were Caucasian, 10% were South Asian and 15% were Black. Although it might be expected that facial reddening is perceived differently in faces of different ethnicities, Stephen et al. [25] found that the effect of facial reddening on apparent health of human faces does not differ according to the ethnicity of the face being judged, or of the observer.

### Questionnaire measures

The online survey also included the following questionnaire measures:

#### The Patient Health Questionnaire-15 (PHQ-15)

The PHQ-15 [34] is a brief, self-administered measure of the frequency and severity of fifteen of the most commonly experienced physical symptoms, which account for more than 90% of symptoms seen in primary care [34]. Participants rated how bothered they had been by fifteen common physical symptoms such as headaches, stomach pain, dizziness and fatigue over the past four weeks on a scale from one (‘not bothered at all’) to two (‘bothered a lot’). Scores range from zero to thirty, with high scores indicating a high degree of physical symptom experience. The PHQ-15 has good internal consistency [34], [35] and test-retest reliability [36].

#### The Rosenberg Self-Esteem Scale (RSE)

The RSE [37] is a widely used measure of self-esteem consisting of ten statements relating to feelings about the self such as, “I feel that I’m a person of worth, at least on an equal plane with others” and, “I certainly feel useless at times”. Respondents rated the degree to which they agreed with each item (in accordance with how they generally feel) on a scale from one (strongly agree) to four (strongly disagree). Scores range from ten to forty with high scores indicating high self-esteem. The RSE has high reliability and internal consistency [38].

### Study design and procedure

Participants in the positive and negative affect groups attended one forty minute testing session. First, participants completed a state version of the PANAS (indicating to what extent they felt each emotion ‘right now’). Next, participants completed self and stranger versions of the FHJ task, the self-esteem Implicit Association Test (IAT) [12], and state versions of the RSE and PHQ-15 (participants responded according to how they felt at the present moment).

### The face health judgement task

#### Materials and image manipulation

Before attending the main testing session, participants met the experimenter to get their photograph taken. Participants were seated in a windowless testing cubicle, against a grey background and photographed with a neutral facial expression, without eyewear or hair covering their face. The testing cubicle was lit with fluorescent bulbs, and photographs were taken using a Nikon D50 digital SLR camera, with a flash.

Each participants’ original photograph was cropped to an oval containing only their face (with hair, clothes and background eliminated) and pasted onto a grey background (see Figures). Following Stephen et al. [25] (cross-cultural study), the amount of redness/greenness in the skin was manipulated by adding/subtracting values of A to/from the original photograph, in the CIE LAB colour space, using Matlab. The CIE LAB colour space consists of three axes representing the amount of lightness-darkness (L), redness-greenness (A) and blueness-yellowness (B) in an image. This colour space is device independent and LAB colour values approximate human vision. The eyes were left out of the colour transform (so that the whites and iris’ of the eyes remained unchanged). Although Stephen and colleagues colour calibrated their images to ensure that their photographs were a true representation of actual skin colour. We did not do so, because we were interested in where participants placed themselves on the red-green axis, rather than measuring participants’ ability to detect their actual, unaltered skin colour. 125 face versions were produced for each participant, which varied from green-tinged (−17 values of A) to red-tinged (+14 values of A) in steps of 0.25. In a pilot study, fifty nine participants rated fourteen altered, and the original versions of their own and a stranger’s face ranging from green (−15 values of A) to red-tinged (+10 values of A) on a scale from zero (very unhealthy) to nine (very healthy). Red-tinged face versions were rated as more healthy looking than green-tinged versions, with healthiness ratings highest for face versions with +1.5 (*M* rating = 6.19) to +2.5 (*M* rating = 6.41) values of A. On average, the red versions of participants own, and stranger’s faces were rated as more healthy looking than green versions (*t*(57) = 5.98, *p*<.001, *d* = 1.58 and *t*(57) = 3.17, *p* = .002, *d* = .84, respectively).

If participants gave their consent, their photograph was used to make a ‘stranger’ version of the face health judgement task to be viewed by other participants. Photographs of seven participants from the current study and twenty four participants from a pilot study were presented to participants in the stranger versions of the task. The stranger’s face allocated to each participant was decided by matching participants in age, gender and as closely as possible in initial facial redness (A values; *M* difference = .06) to another face. A total of thirty one faces were allocated to be used in the stranger versions of the task and six of these faces were judged by more than one participant. Participant and stranger faces were not matched in attractiveness, however, post-hoc ratings of attractiveness (from one: not at all attractive, to ten: extremely attractive) by nine independent raters suggested that participant and control faces were equivalent in attractiveness. Attractiveness ratings for the participants’ faces ranged from 2.56 to 6.89 (*M* = 4.41, *SD* = 1.09). Ratings for the faces used as strangers ranged from 2.89 to 7.33 (*M* = 4.80, *SD* = 1.21). The average difference in attractiveness between participants own face and their allocated stranger was similar in the high NA group (*M* difference = .28, *SD* = 1.41) and the high PA group (*M* difference = .48, *SD* = 1.51, *t*(38) = −.43, *p* = .67).

Task instructions and face stimuli were presented on a computer monitor (faces were presented in a 15×20 cm frame), using E-prime software (Psychology Software Tools Inc., Pittsburgh, PA, USA) and participants responded using a computer keyboard.

#### Procedure

Participants were seated approximately 60 cm in front of the computer monitor. On each trial, participants were presented with a single face version and asked “Do you currently feel more or less healthy than this face?” Participants were instructed to base their decision on the skin tone of the face, rather than any other aspect of it and responded by pressing “M” (more) or “L” (less) on the computer keyboard. First, participants completed four practice trials, showing the −15 (very green), −5.5 (slightly green), +5.5 (slightly red) and +15 (very red) faces.

50% thresholds were then determined using a computerised forced choice adaptive procedure; that is, the face version presented on each trial depended on the participant’s responses on previous trials. The selection of face version on each trial was made using Parameter Estimation by Sequential Testing (PEST) [39] which is an adaptive method of quickly and efficiently estimating psychophysical parameters. The PEST procedure began by presenting a noticeably green face (−15 values of A). A Wald [40] sequential likelihood-ratio test was used to determine when to change face version [39]. Initial step size was set to 4 (that is, a change in A values = 1).

Subsequent step size was determined using the following rules:

1. On every reversal of direction, the step size is halved (unless it follows a double, see rule 3).
2. The second step in a given direction is the same size as the first.
3. If a sequence of three steps in the same direction occurs, then double the step size.
4. The fourth and subsequent steps in the same direction are each double the step size of their predecessor (with a maximum step size set to 8 equivalent to a change in A = 2).
5. After each reversal that follows a double, no change to the step size.
6. End when the minimum step size is reached (set to 1, that is, a change in A values = 0.25).

Because pilot testing suggested that excessive redness to the skin makes faces look unhealthy, step direction also depended on the amount of redness in the face version previously presented. Up to +11 values of A, “more” responses >50% led to a step up (i.e., the selection of a slightly redder face). Above +11 values of A, “more” responses >50% led to a step down by the maximum step size (i.e., the selection of a greener face version). This was to prevent 100% “more” responses at the extreme red end of the colour spectrum which would make it impossible to find the participants’ threshold.

To maintain variability and stop the task becoming too difficult, 75% of trials were adaptive staircase trials, while the remaining 25% were dummy trials on which a face version between −17 and +14 values of A was randomly selected. The adaptive staircase task took between one to fifteen minutes to complete, depending on how long the PEST algorithm took to determine the participants 50% threshold (five minutes on average, *M* no. of trials = 77, *SD* = 50).

Participants completed two versions of the face health judgement task, one in which they viewed their own face and one in which they viewed another person’s face. The participant confirmed that they were not familiar with the stranger. To clarify, in the self version of the task, participants judged whether they currently felt more or less healthy than each version of their own face. In the stranger version of the task, participants judged whether they currently felt more or less healthy than each version of the stranger’s face. Self/stranger task order was counterbalanced between participants to control for any comparison effects.

### The self-esteem implicit association test (IAT)

#### Materials

Instructions and stimuli were presented on the computer monitor using e-prime software. Following Greenwald and Farnham [13], five self (myself, mine, me, my, self), other (other, them, their, they, them), positive (rainbow, happy, smile, warmth, joy) and negative (pain, death, poison, grief, agony) words were used as stimuli.

#### Design and procedure

The IAT comprised the usual five-block procedure [41], with sixty trials in each critical block (see Table 1). Critical block order was kept constant to minimize the effect of procedural variations on the measurement of individual differences in self-esteem [42], [43].

**Table 1:** The five block IAT procedure^1^.

Block	Press the ‘Z’ (left) key for:	Press the ‘M’ (right) key for:	Purpose
1 (20 practice trials)	Self	Other	Learning the conceptdimension
2 (20 practice trials)	Positive	Negative	Learning the attributedimension
*3 (20 practice trials,* *60 critical trials)*	*Self or positive*	*Other or negative*	*Combined block 1*
4 (40 practice trials)	Other	Self	Learning to switch the spatiallocation of the concepts
*5 (20 practice trials,* *60 critical trials)*	*Other or positive*	*Self or negative*	*Combined block 2*

1 Critical blocks are shown in italics. The IAT effect is computed as the difference in mean response latency between Blocks three and five. Including forty practice trials in Block four is recommended to compensate for the extraneous influence of the order of the combined blocks [48].

Participants remained seated approximately 60 cm from the computer monitor and were presented with one of the self, other, positive or negative words in the centre of the computer screen. Participants were instructed to press either a left (‘Z’) or right (‘M’) response key on the computer keyboard to rapidly categorise the word as self or other (Blocks one and four), positive or negative (Block two), self/positive or other/negative (Block three), other/positive or self/negative (Block five). Labels at the top left and top right hand corners of the screen indicated which category went with the left or right response key. In Blocks three and five, which involved categorising both concept (self/other) and attribute words (positive/negative), concept trials were alternated with attribute trials. If the participant made an incorrect response a red X appeared in the centre of the screen and participants were required to make the correct response before moving on to the next trial. Participants were instructed to keep their index fingers on the ‘Z’ and ‘M’ keys to enable a rapid response. The IAT took approximately five minutes to complete. IAT data were processed using the improved scoring algorithm D_1_ measure, which is an effect size comparable to Cohen’s *d* [44].

### Data analysis

To determine whether face health judgement differed between participant groups, a 2(Group: NA/PA)×2(FHJ task version: self/stranger) mixed design ANOVA was performed with 50% thresholds (determined by the adaptive staircase program) as the dependent variable and initial facial redness and self/stranger task order included as covariates. 50% thresholds were also correlated with scores on the PHQ-15, RSE and IAT.

## Results

Table 2 shows descriptive statistics for PHQ-15, RSE and IAT scores in the positive and negative affect groups. Groups did not differ in initial facial redness (*t*(38) = .64, *p* = .52, *d* = .21, *M* positive affect group = 13.63, *M* negative affect group = 15.01).

**Table 2:** Descriptive statistics for scores on the PHQ-15, RSE and IAT in each group.

		*PHQ-15 (state)* **	*PHQ-15 (trait)* **	*RSE (state)* ***	*RSE (trait)* ***	*D_1_ (IAT)* **
Positive Affect Group	*M*	5.25	6.80	33.60	32.25	.78
	*SD*	*3.94*	*4.29*	*4.39*	*14.55*	.*36*
Negative Affect Group	*M*	9.70	11.20	25.25	24.00	.50
	*SD*	*5.30*	*5.30*	*5.37*	*6.02*	.*34*

** = difference between PA and NA groups significant at *p*<.01.

*** = difference between PA and NA groups significant at *p*<.001.

### Did the point of subjective equality differ between participant groups?

**Figure 1:**
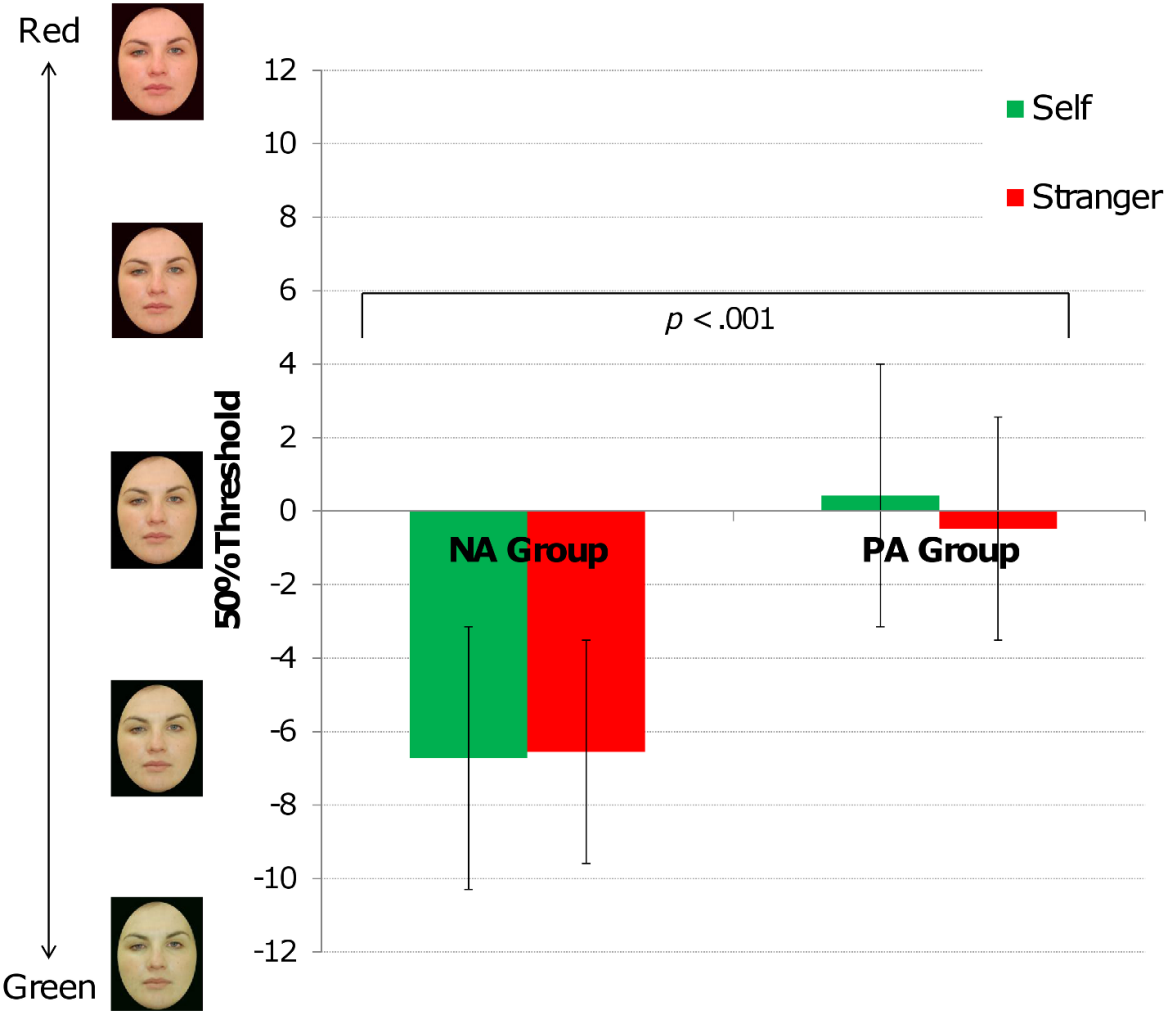
Average 50% thresholds on the self and stranger face health judgement task. The x-axis shows 50% thresholds ranging from −12 (very green) to 12 (very red). In both versions of the task, participants indicated how they judged themselves in comparison either to different versions of their own face or to different versions of a stranger’s face. Self 50% thresholds indicate how participants perceived themselves. Stranger 50% thresholds indicate how participants judged themselves in comparison to the stranger. The negative affect group (unhappy participants, left hand side) had significantly lower 50% thresholds on both versions of the face health judgement task compared to the positive affect group (happy participants, right hand side). That is, the negative affect group judged themselves as equivalent to greener, less healthy looking versions of their own and a stranger’s face. Error bars reflect ±1 standard error of the mean.

There was no effect of face health judgement task version (*F*(1,36) = .02, MSE = 15.26, *p* = .90, *d* = .09) and no interaction between face health judgement task version and group (*F*(1,36) = .36, MSE = 15.26, *p* = .55). There was, however, a main effect of group (*F*(1,36) = 11.96, MSE = 77.83, *p* = .001, *d* = .77). For the negative affect group, the point of indifference fell at a greener, less healthy looking face compared to the positive affect group, on both versions of the face health judgement task. There was no effect of self/stranger task order (*F*(1,36) = 1.64, MSE = 77.83, *p* = .21, *d* = .30) and no other main effects or interactions were significant (*p*’s>.47). Figure 1 illustrates these results.

### How was FHJ related to positive and negative affect, subjective perceptions of health, and self-esteem?

Negative affect scores in the positive affect group were not normally distributed. State negative affect scores were positively skewed; most participants in the positive affect group reported low negative affect. These data remained non-normally distributed after transformation attempts and so were analysed using non-parametric correlations (to control for multiple correlations, the significance level was lowered to *p* = .01).

**Figure 2:**
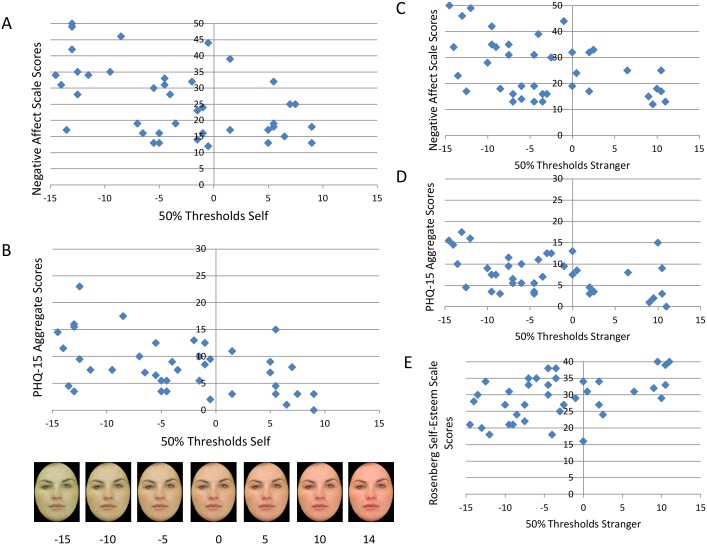
Correlations between scores on the questionnaire measures and 50% thresholds on each version of the face health judgement task. **A:** Illustrates the negative relationship between 50% thresholds on the self version of the task and negative affect scale scores. **B:** Illustrates the negative relationship between 50% thresholds on the self version of the task and PHQ-15 scores. As state and trait PHQ-15 scores were strongly correlated (*r* = .84, *p*<.001), aggregate scores were calculated for the purposes of illustration. **C and D:** Illustrate the negative relationship between 50% thresholds on the stranger version of the task and negative affect scale scores and PHQ-15 scores. Higher negative affect and physical symptom reports were associated with lower 50% thresholds on both versions of the task (less healthy self-image/unfavourable social comparisons). **E:** Shows the significant positive relationship between stranger face health judgement and self-esteem; high self-esteem was associated with more favourable social comparisons.

Figure 2 illustrates significant correlations. 50% thresholds on the self face health judgement task were correlated negatively with trait negative affect (*r* = −.50, *p*<.001), tended to be correlated negatively with state negative affect (*r* = −.36, *p* = .02) and tended to be correlated positively with positive affect (trait, *r* = .36, *p* = .03, and state, *r* = .29, *p* = .07). 50% thresholds were also correlated negatively with PHQ-15 scores (trait, *r* = −.47, *p* = .002, and state, *r* = −.44, *p* = .01) and tended to be correlated positively with state (but not trait) RSE scores (*r* = .34, *p* = .03). 50% thresholds were not correlated with IAT D_1_ scores (*r* = −.02, *p* = .88).

50% thresholds on the stranger face health judgement task were correlated negatively with trait negative affect (*r* = −.42, *p* = .01). The correlation with state negative affect was in the expected direction, but did not reach significance (*r* = −.26, *p* = .10). 50% thresholds on the stranger face health judgement task were not correlated with trait or state positive affect (*p* = .30 and *p* = .17, respectively), but were correlated negatively with PHQ-15 scores (trait *r* = −.51, *p* = .001, and state, *r* = −.42, *p* = .01) and were correlated positively with state RSE scores (*r* = .40, *p* = .01). The correlation with trait RSE scores was in the expected direction but did not reach significance (*r* = .31, *p* = .05). 50% thresholds were not correlated with IAT D_1_ scores (*r* = .07, *p* = .68). Self and Stranger 50% thresholds were also correlated (*r* = .75, *p*<.001).

## Discussion

We developed a novel face health judgement task to investigate individual differences in health perception in groups of participants characterised by high positive versus high negative affectivity. Based on previous research with student samples [16], [17], [24], we expected the positive affect group to show enhancement effects, i.e., judge themselves as equivalent to healthier looking versions of their own and stranger’s faces. The negative affect group were expected to either lack these enhancement effects or show derogation in self-image, i.e., judge themselves as equivalent to less healthy looking versions of their own and stranger’s faces. The results were broadly in line with our predictions; the positive affect group had higher 50% thresholds on both the self and stranger version of the face health judgement task compared to the negative affect group. These between group differences were driven by derogation in the negative affect group, rather than enhancement in the positive affect group, however.

Previous studies have found that students have enhanced perceptions of self-attractiveness, especially those high in implicit self-esteem [16], [24] and enhanced perceptions of how trustworthy their face looks [17]. We improved on the paradigms used previously to measure self-image, by manipulating healthiness of skin tone, rather than morphing participants’ faces with other faces (our set of faces did not differ in the degree to which they looked physically similar or dissimilar to the participant) and measured perceptual thresholds using psychometric methods. Using our face health judgement task, we found limited evidence to suggest that the enhancement effects found previously extend to healthiness of self-image. Although state and trait positive affect tended to be positively associated with healthiness of self-image, on average, the positive affect group were relatively unbiased in their face health judgement. The apparent lack of enhancement in the positive affect group could be due to limitations of our image manipulation methods. We are confident that our colour manipulations had the desired effect on how healthy our faces looked. However, we did not colour calibrate our original photographs and as a result, there is a possibility that participants looked slightly healthier in their original photographs than in reality. If so, 50% thresholds around zero would represent small enhancement effects. We do not think that the lack of colour calibration can account for our finding of individual differences in face health judgement, as any inherent bias in the colour of our original photographs would have affected both groups in the same way. All photographs were taken using the same camera, using the same settings, and under identical lighting conditions. However, colour calibration would have enhanced the interpretability of our findings and would have allowed us to more accurately measure perceptions of self-image. The possibility remains, however, that not all participants have an enhanced perception of their self-image, even if they are characterised by high positive affect; in Clerkin & Teachman’s [24] study students without symptoms of body dysmorphia accurately identified their un-altered image out of a line-up of attractive and unattractive morphs.

In previous studies, individuals with eating disorders and symptoms of body dysmorphia have been found to have unflattering perceptions of their self-image, in line with their specific concerns [15], [24]. Our findings suggest that experiencing more generalised negative affectivity, rather than having specific concerns about one’s appearance, also affects the perception of self-image. The link between affectivity and healthiness of self-image could be mediated to some extent by physical symptom perception. The negative affect group reported a higher frequency and severity of physical symptoms, and symptom reports were correlated with healthiness of self-image.

Although cause and effect is yet to be established, if participants characterised by high negative affectivity see themselves as looking less healthy than they do in reality, it could lead them to experience further negative affect, forming a vicious cycle; Zell and Balcetis [33] note that lower-level processes are often the building blocks upon which higher level cognition and action are based.

It is notable that the majority of our participants were female. Previous studies have found gender differences in self-image; for example Gentile et al. [45] found evidence of lower physical appearance self-esteem in females, perhaps because the media promote particularly high standards for female appearance [45]. In addition, females tend to report higher negative affect than men [4] and more physical symptoms [2], [46]. What is more, correlations between negative affect and physical symptom ratings differ between men and women [47]. It is possible, therefore, that the link between negative affect and unflattering face health judgement is stronger in, or even unique to females. Gender differences in the link between negative affect and face health judgement could be a promising avenue for future research.

Our findings suggest that negative affect also modulates how people judge themselves against others. The negative affect group judged themselves as equivalent to less healthy looking versions of stranger’s faces compared to the positive affect group. These results are consistent with previous findings that individuals characterised by negative affect compare themselves unfavourably to others [29]. We have shown that this extends to judgements of healthiness. As mentioned in the introduction, self-other comparisons are thought to shape our self-evaluations [30]–[32] and can even affect how we perceive our own face [33]. Judging oneself as less healthy in comparison to others could, therefore, maintain a negative self-concept and self-image, and again, contribute to a vicious cycle of negative affect and altered perception. In line with this idea, healthiness of self-image tended to be associated with explicit (but not implicit) self-esteem.

The attractiveness of another person’s face has previously been found to moderate the effect of social comparison on self-image, that is, comparing oneself with a more attractive person has a negative impact on one’s self-image [31]. We did not match participants and ‘strangers’ based on attractiveness. However, on average, post hoc attractiveness ratings for the participant and stranger faces were similar. In addition, differences in attractiveness between each participant and their allocated stranger face were small and similar in the PA and NA groups. However, the possibility remains that small discrepancies in self-stranger attractiveness influenced the results (i.e., comparing oneself to a more/less attractive stranger could affect one’s mood). Although there was no evidence of a carry-over effect of social comparisons during the stranger version of the FHJ task (there was no significant effect of self/stranger task order), in future studies, each participant should be matched to an equivalently attractive stranger to rule out his confound.

The face health judgement task has ecological validity as a perceptual measure of well-being and could potentially be used to corroborate self-report measures. As noted in the introduction, we often use facial appearance as a cue to health and are used to seeing more and less healthy looking versions of ourselves and other people. Both versions of the face health judgement task could potentially be used as measures of well-being, subject to further validation, but the stranger version would be more practical to develop and administer. We are currently investigating whether self and stranger face health judgement is sensitive to laboratory manipulations of mood. To further validate the task as a perceptual measure of well-being, it will be necessary to establish convergent and discriminant validity and test-retest reliability with larger samples of participants. A variation of the face health judgement task could also be developed to alter perceptions of self-image in groups characterised by negative affect. Via feedback, participants could be trained to be more accurate in their self-perceptions, which could heighten their mood and break the cycle of negative affect and perceptual bias.

Using a novel face health judgement task, we have shown that unhappy people have reduced healthiness of self-image compared to happy people and that individual differences in positive and negative affect influence how people judge their healthiness against others. Face health judgement could be used as an indicator of well-being, or even a target of interventions for negative affect groups.
